# Agreement in non-cycloplegic and cycloplegic refraction between a photoscreener and a calibrated autorefractor

**DOI:** 10.1186/s12886-024-03375-z

**Published:** 2024-03-25

**Authors:** Piotr Kanclerz, Katarzyna Przewłócka, Robert W. Arnold

**Affiliations:** 1 Helsinki Retina Research Group, University of Helsinki, Helsinki, Finland; 2 Hygeia Clinic, Department of Ophthalmology, Gdańsk, Poland; 3 Alaska Blind Child Discovery, Anchorage, AK USA

**Keywords:** Autorefractometry, Cycloplegic measurements, Photoscreeners, Refractive errors

## Abstract

**Introduction:**

Photoscreeners have been shown to provide excellent measurements of the refractive error. However, whether they could be used for assessing cycloplegic refraction has not been examied. This study aimed to evaluate the agreement between cycloplegic and non-cycloplegic measurements obtained using a photoscreener and stationary autorefractor, respectively.

**Methods:**

This study included all patients undergoing routine ophthalmic examination at the Hygeia Clinic (Poland) from June to July 2022. Each patient underwent non-cycloplegic and cycloplegic refraction assessments using the 2WIN photoscreener (Adaptica SRL, Padova, Italy) and an ARK-1 stationary autorefractor ARK-1 (Nidek Co Ltd., Tokyo, Japan), respectively. Each pair of assessments was conducted in random order, and all values were determined at a vertical distance of 12 mm. The agreement between cycloplegic and non-cycloplegic measurements was assessed using paired *t*-tests, Bland-Altman and ABCD ellipsoids.

**Results:**

This analysis included 82 patients, of which 52 were female. Their mean age was 34.39 ± 13.13 years. The non-cycloplegic spherical equivalent (SE) did not differ significantly between the 2WIN (− 1.22 ± 2.45) and ARK-1 (− 1.19 ± 2.96) devices (*p* = 0.580). However, the cycloplegic SE values demonstrated more negative values with the 2WIN device (− 1.13 ± 2.19) than with the ARK-1 device (− 0.75 ± 3.03; *p* = 0.007). The non-cycloplegic and cycloplegic measurements were strongly correlated between the devices (*r* = 0.9473 and 0.9411, respectively). However, the correlation between their cycloplegic shifts in SE was low (*r* = 0.2645). Ellipsoid refraction aligned better non-cycloplegic (ARK-1 = 1.00; 2WIN = 1.74) than with cycloplegic refraction (ARK-1 = 1.43; 2WIN = 1.90).

**Conclusion:**

While the cycloplegic measurements obtained with the 2WIN photoscreener were strongly correlated with those obtained with the ARK-1 stationary autorefractor for most of the analyzed parameters, they should not be considered interchangeable.

## Introduction

Photoscreeners have been designed to detect risk factors for amblyopia, rather than amblyopia or structural ocular alterations [1]. Photoscreening is currently a recognized method for vision screening in children aged 3–5 years and uncooperative older children. The American Academy of Pediatrics supported the use of photoscreening in its policy statement [2, 3]. Vision screening should generally be performed several times during a child’s formative years. It should have high specificity in younger children and high sensitivity in older children [4].

Photoscreeners use a flash camera with an acute flash-to-patient lens angle of approximately one degree such that refractive errors, a risk factor for amblyopia can be detected by a light crescent encroaching on the otherwise uniform red pupil reflex; the greater the light crescent, the greater the refractive defocus [5]. Current commercially available photoscreeners use infrared light and internal computational interpretation to estimate binocular refractive error, pupil size, and pupillary distance. The advantages of the photoscreener design include instantaneous assessment and convenience for testing children.

There are currently several photoscreener available on the market, include the S12 (Plusoptix, Nurnberg, Germany), Blinq (RebiScan, Boston, MA, USA), 2WIN (Adaptica SRL, Padova, Italy) [6] and Spot (Welch Allyn, Auburn, NY, USA). Some other devices, such as the iScreen (iScreen Vision, Inc, Cordova, TN, USA) [7] and GoCheckKids (Gobiquity, Nashville, TN, USA) [8, 9], use visible light with central reading centers. The Blinq (Rebiscan, Boston, MA, USA) device screens for ocular misalignment but not refraction. The 2WIN device is a portable binocular refractometer and vision screener commonly used in pediatric eye care. Overall, the 2WIN device offers the advantages of portability, efficiency, objectivity, non-invasiveness, and comprehensive vision assessment, making it a valuable tool in pediatric eye care for screening refractive errors and assessing vision in children.

The precision of photoscreeners in assessing the magnitude of the refractive error has improved over the last two decades [10–12]. Some studies have even postulated that non-cycloplegic photorefraction has acceptable accuracy and advantages over cycloplegic retinoscopy [11]. Potentially, using photoscreeners to evaluate cycloplegic refraction has the potential to enhance the standard of eye care, especially in underserved and distant locations.

Agreement studies are critical for clinical decision making when selecting devices or methods to assess refractive error assessment. If non-cycloplegic and cycloplegic refractions show a high level of agreement between the 2WIN and ARK-1 devices, it instills confidence in using either device interchangeably. In contrast, inconsistent or discordant measurements between the devices may lead to variations in the refractive correction prescribed, potentially impacting visual outcomes for patients. Therefore, this study aimed to evaluate the agreement between cycloplegic and non-cycloplegic measurements obtained with the 2WIN photoscreener and the conventional ARK-1 stationary autorefractor.

## Methods

This study included all adults undergoing routine ophthalmic examination at the Gdańsk and Elbląg branches of the Hygeia Clinic between June and July 2022. Patients with cataracts, glaucoma, prior ocular surgery, or suffering from any other ocular diseases were excluded. This study adhered to the tenets of the Declaration of Helsinki, and written informed consent was obtained in all cases. The study protocol was approved by the local bioethical committee (Komisja Bioetyczna przy Izbie Lekarskiej w Gdańsku; approval no.: KB-40/22).

**Fig. 1:**
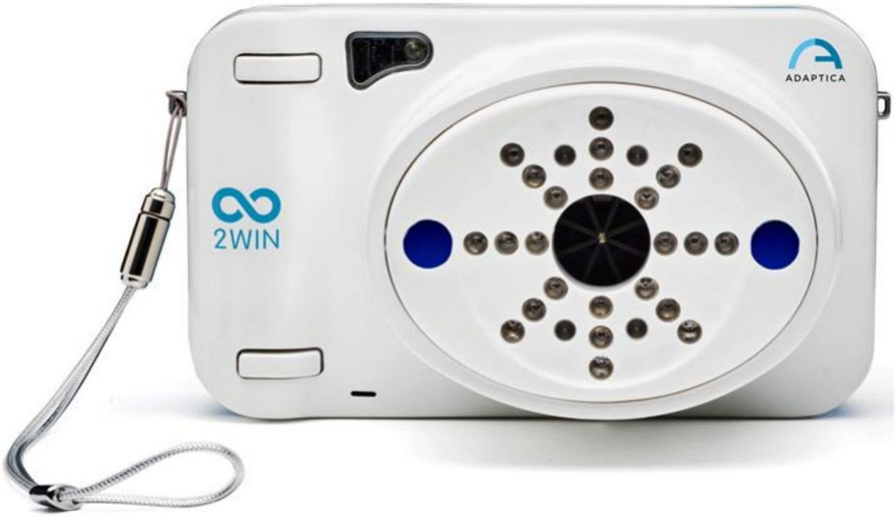
A photo of the Adaptica 2WIN (Adaptica SRL, Padova, Italy) photoscreener

The 2WIN (Fig. 1) is a hand-held vision screener that measures both eyes simultaneously at a distance of one meter. It has a measurement range of −15.00 to +15.00 diopter (D) sphere and up to 5.00 D cylinder. It provides not only objective refraction measurements but also allows for analyzing the corneal reflexes, performing dynamic pupillometry, and assessing the lens centering of glasses. The Nidek ARK-1 is a stationary device that combines autorefraction, keratometry, and pupillography. It uses the Scheiner disc principle [13] and a large pupil zone imaging method and has a measurement range of −30.00 to +25.00 D sphere and up to 12.00 D cylinder. The autorefractor incorporates a super luminescent diode that provides a clearer and sharper image compared to older designs and a highly sensitive charge-coupled device that is stated to allow the autorefractor to perform measurement in densely cataractous eyes.

**Fig. 2:**
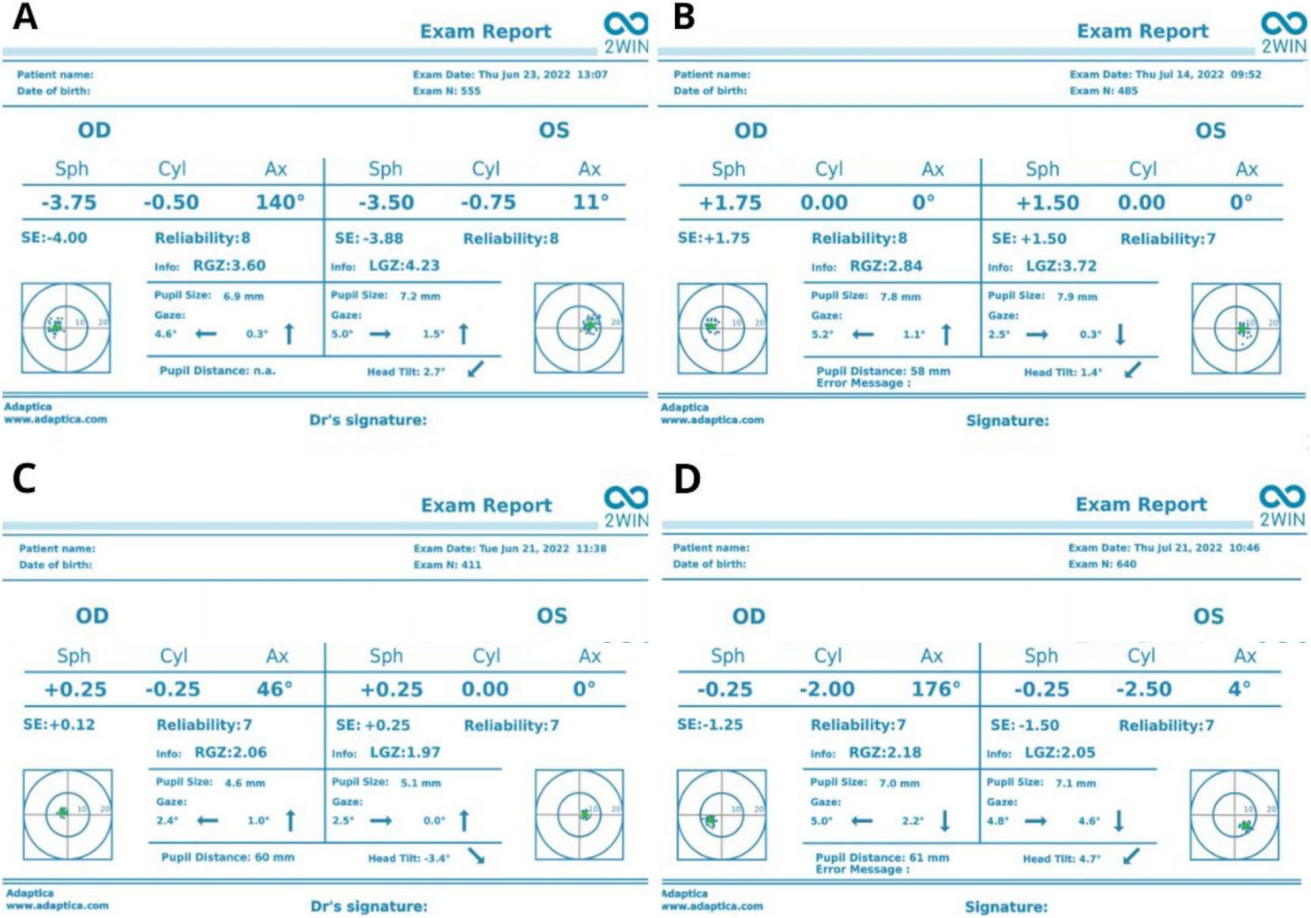
Selected results obtained from the 2WIN device in patients with **(A)** myopia; **(B)** hyperopia; **(C)** emmetropia; **(D)** astigmatism

Measurements were conducted with the two devices in random order; this study only used the results for the right eyes. All examinations were conducted between 08:00 and 15:00 in similar dim light conditions (under 10 lm). For the 2WIN measurements, the patient was instructed to keep their head vertical and look at the device at a distance of one meter. The results were considered valid if the quality mark on the scan was assessed as OK; up to three measurements were taken with each device until a valid result was obtained, and cases with insufficient imaging quality were noted. For each device, the magnitude of refractive error (sphere, cylinder and axis) and the pupillary diameter were recorded. Sample results obtained with the 2WIN device have been presented in Fig. 2. Visual acuity with manifest refraction was also recorded with the finest acuity truncated at fraction 1.0 (logarithm of the minimum angle of resolution [logMAR] = 0.0). The manifest refraction from the phoropter was recorded. Following non-cycloplegic measurements, every patient had both eyes instilled with two sets of 1% tropicamide eye drops separated by five minutes [14]. After 30 minutes, to allow mydriasis to develop, the measurements were repeated with the two devices in random order. For non-cycloplegic measurements, the  patient’s age was entered into 2WIN; for cycloplegic measurements, an age of 60+ years was used to minimize the influence of accommodative compensation. All refraction measurements were taken at a vertex distance of 12 mm.

Myopia was defined as a myopic refractive spherical equivalent (SE) of ≥0.50 D in manifest refraction. SE was calculated as a sum of the sphere and half of the cylinder. Hyperopia was defined as a hyperopic SE greater than +1.0 D. The ABCD ellipsoid is a single unit metric reflecting the comparable ability to resolve visual acuity blur by two spectacle refractions with a perfect match scoring 0.0, one blurred line scoring 1.0, three blurred lines 2.0, and six blurred lines scoring 3.0 [15]. The “ABCD composite” obtained combines a simplified cylinder J0 vs. J45 component with the SE grade resulting in a single combined measure of the spherocylinder [16].

The results are presented as the mean ± standard deviation. Cylinder vector values were calculated as recommended elsewhere [17]; the polar values along the zero-degree meridian (J0) and the 45° meridian are presented (J45). The normality of the data was confirmed using the Kolmogorov–Smirnov test, which showed a normal distribution. A two-tailed *t-*test was used to compare normally distributed data. The linear correlation between the measurements of the two devices was assessed using Pearson’s product-moment correlation coefficients (*r*); 0 ≤ *r* < 0.3 was considered weak positive, 0.3 ≤ *r* < 0.7 was considered moderate positive, and 0.7 ≤ *r* ≤ 1.0 was considered strong positive [18]. A sample size of 54 eyes was estimated to detect a 0.05 D difference in refraction between the devices based on a standard deviation of 0.1 D, a power of 95%, and a significance level of 5%. A *p*-value of <0.05 was considered as statistically significant. The statistical analyses were performed using Medcalc Software (version 14; Medcalc Software Ltd., Ostend, Belgium) and IBM SPSS Statistics (version 28; IBM Corp., Armonk, NY, USA).

## Results

Eighty eight adults were examined with both devices; it was impossible to obtain results with the 2WIN device in five cases and in one case with the ARK-1 device in one case. Therefore, eighty-two patients were included in the analysis, of which 52 were female and 31 were male. Their mean age was 34.4 ± 13.1 years (range: 18–62 years). With the logMAR chart, their mean uncorrected visual acuity was 0.38 ± 0.37, and their best corrected visual acuity was 0.05 ± 0.12. Their mean manifest SE refraction was −0.95 ± 2.70 D, cylinder was −0.42 ± 0.82 D, and axis was 62.37° ± 71.10° degrees. Fifteen patients were classified as emmetropes, 44 as myopes, and 23 as hyperopes.

The non-cycloplegic SE values did not differ significantly between 2WIN (−1.22 ± 2.45 D) and ARK-1 (−1.19 ± 2.96) devices (*p* = 0.580). However, cycloplegic SE values were more negative with the 2WIN device (−1.13 ± 2.19) than with ARK-1 device (−0.75 ± 3.03; p *=* 0.007). The non-cycloplegic and cycloplegic measurements did not differ significantly for most of the other parameters (Table 1).

**Fig. 3:**
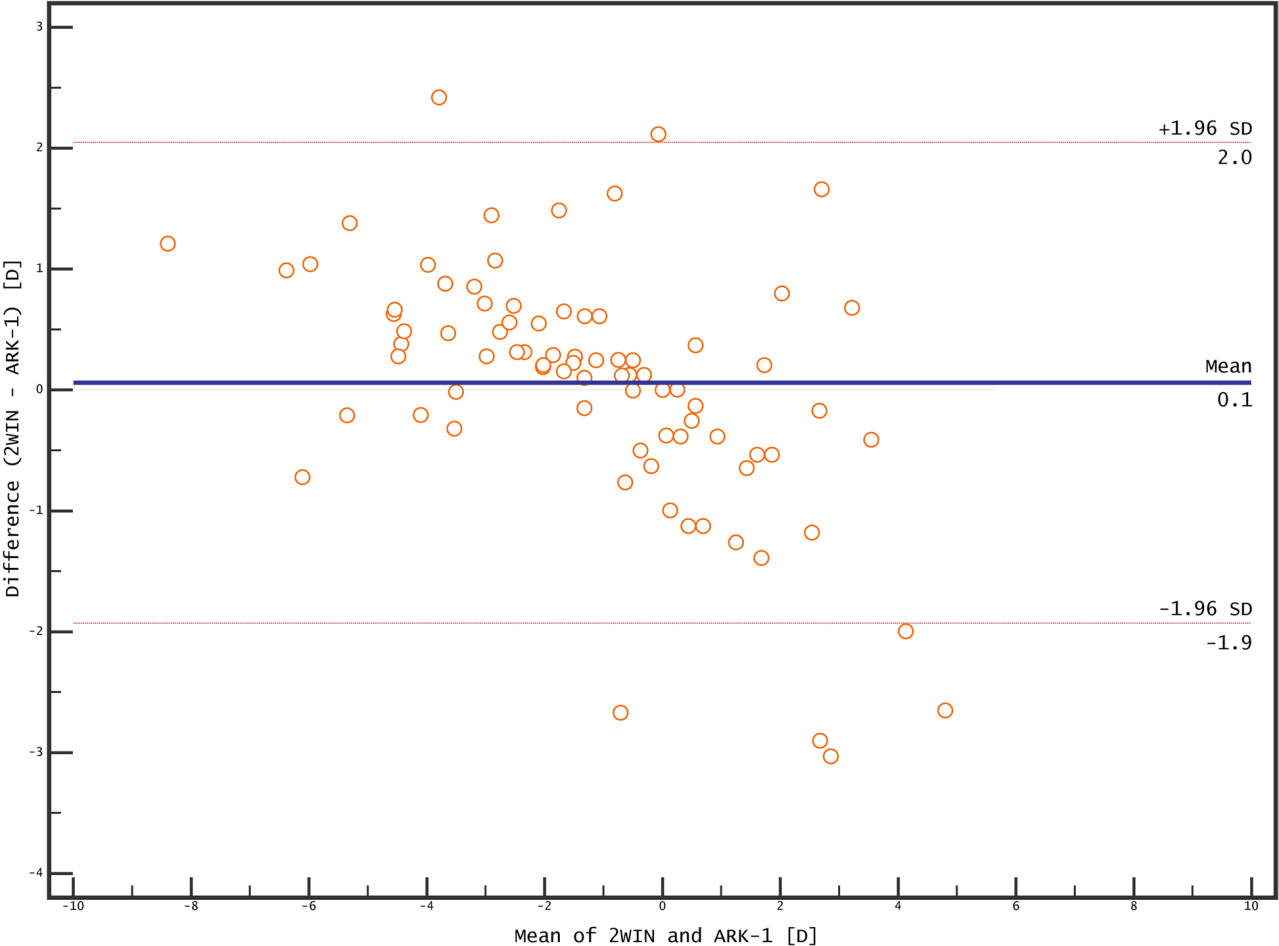
Agreement in non-cycloplegic refraction between 2WIN and ARK-1

**Table 1:** Comparison of the results obtained with Adaptica 2WIN and Nidek ARK-1

Parameter	Adaptica 2WIN	Nidek ARK-1	Difference (2WIN - ARK-1)	95% LOA	Correlation coefficient *r* (*p*)	*p* value
**Dsph**	− 0.80 ± 2.46	− 0.83 ± 2.97	0.03 ± 0.98	− 1.89 to 1.95	**0.95 (** ***p*** **< 0.0001)**	**0.58**
**Dcyl**	− 0.75 ± 0.75	− 0.82 ± 0.81	0.07 ± 0.38	− 0.67 to 0.81	**0.89 (** ***p*** **< 0.0001)**	**0.56**
**Axis**	78.40 ± 68.35	88.18 ± 67.79	− 9.78 ± 8.45	−26.09 to − 6.53	**0.37 (** ***p*** **< 0.0006)**	**0.34**
**J0**	0.19 ± 0.43	0.22 ± 0.47	− 0.03 ± 0.18	− 0.38 to 0.32	**0.92 (** ***p*** **< 0.0001)**	**0.72**
**J45**	0.01 ± 0.26	0.00 ± 0.27	0.01 ± 0.15	− 0.28 to 0.30	**0.83 (*p* < 0.0001)**	**0.69**
**SE**	− 1.18 ± 2.46	− 1.24 ± 2.94	0.06 ± 1.00	−1.90 to 2.02	**0.95 (*p* < 0.0001)**	**0.89**
**PD**	63.05 ± 3.54	63.33 ± 3.62	− 0.28 ± 1.69	− 3.59 to 3.03	**0.89 (*p* < 0.0001)**	**0.64**
**Cyclo Dsph**	− 0.87 ± 2.17	−0.34 ± 3.09	−0.53 ± 1.15	− 2.78 to 1.72	**0.94 (*p* < 0.0001)**	**0.33**
**Cyclo Dcyl**	−0.56 ± 0.63	−0.83 ± 0.78	0.27 ± 0.49	−0.69 to 1.23	**0.73 (*p* < 0.0001)**	**<0.01**
**Cyclo Axis**	75.89 ± 69.50	83.86 ± 0.78	− 8.98 ± 7.95	−24.32 to 6.36	**0.43 (*p* < 0.0001)**	**0.46**
**Cyclo J0**	0.13 ± 0.33	0.19 ± 0.47	−0.06 ± 0.35	−0.75 to 0.63	**0.67 (*p* < 0.0001)**	**0.40**
**Cyclo J45**	0.01 ± 0.20	−0.02 ± 0.26	0.03 ± 0.16	−0.28 to 0.34	**0.78 (*p* < 0.0001)**	**0.48**
**Cyclo SE**	−1.13 ± 2.19	−0.76 ± 3.03	−0.37 ± 1.21	−2.74 to 2.00	**0.94 (*p* < 0.0001)**	**0.57**
**Cyclo PD**	63.03 ± 3.57	63.30 ± 3.60	− 0.27 ± 1.68	− 3.56 to 3.02	**0.90 (*p* < 0.0001)**	**0.58**

*Cyclo* Cycloplegic measurement: *J0* polar value along the zero-degree meridian: *J45* polar value along the 45-degree meridian: *LOA* limits of agreement: *PD* pupillary distance: *SE* spherical equivalent

**Fig. 4:**
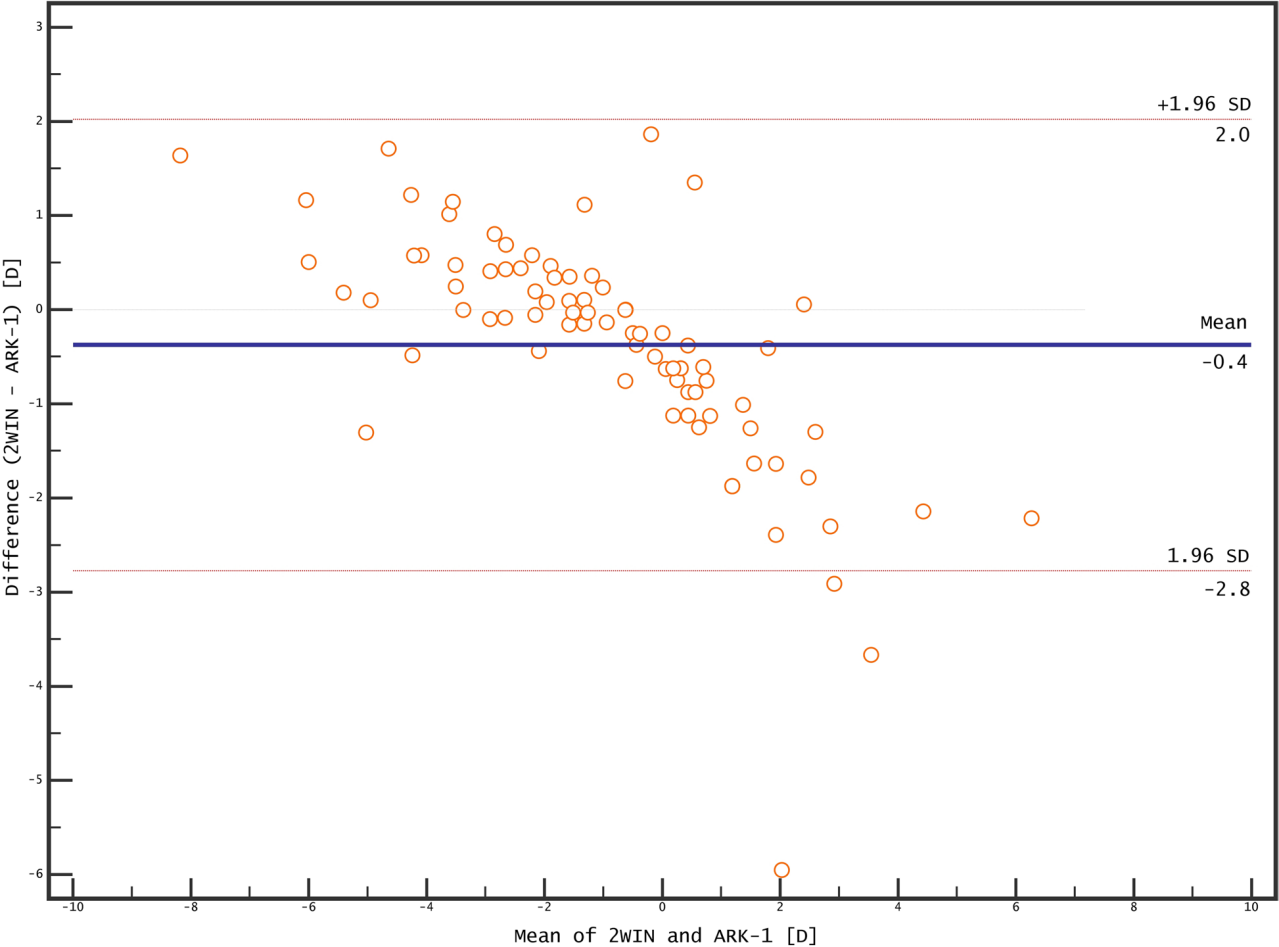
Agreement in cycloplegic refraction between 2WIN and ARK-1

The Bland–Altman plots of the agreement between the 2WIN and ARK-1 devices for non-cycloplegic and cycloplegic refraction are presented in Figs. 3 and 4. The non-cycloplegic cylinder polar values were strongly positively correlated between the devices (*r* = 0.92 and *r* = 0.83 for J0 and J45, respectively). The correlations were weaker for cycloplegic cylinder polar values, but remained positive (*r* = 0.67 and *r* = 0.78 for J0 and J45, respectively)

The 2WIN device underestimated the magnitude of non-cycloplegic SE both in hyperopes (by −0.82 ± 0.44 D; *p* = 0.0006) and in myopes (by 0.60 ± 0.38 D; *p* = 0.0001) compared to the ARK-1 refractor (Table 2). The difference was even more prominent for cycloplegic SE measurements for hyperopes (by −1.64 ± 0.47 D; *p* < 0.0001) but not in myopes (by 0.37 ± 0.37 D; *p* = 0.0002). The SE was strongly correlated between the devices for both non-cycloplegic (*r* = 0.9473, *p* < 0.0001) and cycloplegic (*r* = 0.9411; p < 0.0001) measurements. The cycloplegic shift was significantly smaller with the 2WIN device (0.15 ± 1.06 D) that with the ARK-1 device (0.47 ± 0.53 D); it was weakly linearly correlated between the devices (*r* = 0.1412, *p* < 0.0001).

**Table 2:** Agreement in non-cycloplegic and cycloplegic spherical equivalent refraction among devices according to refractive status

Refractive status	Parameter	Device	Mean value±SD	Mean difference ± SD	95% LoA	Correlation coefficient *r* (*p*)	*p* value
Total (*n* = 82)	Non-cycloplegic SE [D]	2WIN	− 1.18 ± 2.41	0.06 ± 0.42	− 0.76 to 0.88	0.95 (< 0.0001)	0.89
		ARK-1	−1.24 ± 2.94				
	Cycloplegic SE [D]	2WIN	−1.13 ± 2.19	− 0.37 ± 0.41	−1.17 to 0.43	0.94 (< 0.0001)	0.57
		ARK-1	− 0.76 ± 3.03				
	Cycloplegic shift in SE [D]	2WIN	0.05 ± 0.79	−0.43 ± 0.11	− 0.65 to 0.22	0.26 (0.0164)	0.20
		ARK-1	0.48 ± 0.53				
Emmetropes (*n* = 15)	Non-cycloplegic SE [D]	2WIN	−0.37 ± 0.52	−0.06 ± 0.23	0.51 to 0.39	0.57 (<0.0001)	0.03
		ARK-1	−0.31 ± 0.73				
	Cycloplegic SE [D]	2WIN	−0.18 ± 0.50	−0.45 ± 0.17	− 0.78 to − 0.12	0.30 (< 0.0001)	< 0.01
		ARK-1	0.27 ± 0.43				
	Cycloplegic shift in SE [D]	2WIN	0.19 ± 0.64	−0.39 ± 0.26	− 0.90 to 0.12	0.53 (<0.0001)	0.04
		ARK-1	0.58 ± 0.77				
Hyperopes (*n* = 23)	Non-cycloplegic SE [D]	2WIN	1.47 ± 1.42	−0.82 ± 0.44	−1.68 to 0.04	0.66 (<0.0001)	<0.01
		ARK-1	2.29 ± 1.55				
	Cycloplegic SE [D]	2WIN	1.22 ± 1.29	−1.64 ± 0.47	−2.56 to −0.72	0.69 (<0.0001)	<0.01
		ARK-1	2.86 ± 1.71				
	Cycloplegic shift in SE [D]	2WIN	−0.16 ± 1.44	−0.74 ± 0.32	−1.37 to − 0.11	0.32 (<0.0001)	0.13
		ARK-1	0.58 ± 0.54				
Myopes (*n* = 44)	Non-cycloplegic SE [D]	2WIN	−2.80 ± 1.77	0.60 ± 0.38	−0.14 to 1.34	0.94 (<0.0001)	<0.01
		ARK-1	−3.40 ± 1.77				
	Cycloplegic SE [D]	2WIN	−2.63 ± 1.67	0.37 ± 0.37	−0.36 to 1.10	0.94 (<0.0001)	<0.01
		ARK-1	−3.00 ± 1.80				
	Cycloplegic shift in SE [D]	2WIN	0.17 ± 0.55	− 0.23 ± 0.11	− 0.44 to 0.02	0.16 (<0.0001)	0.31
		ARK-1	0.40 ± 0.43				

Cycloplegic shift was defined as the difference between cycloplegic spherical equivalent (SE) and non-cycloplegic SE refraction

*LOA* limits of agreement: *SE* spherical equivalent

The ABCD ellipsoid method compared the manifest refraction with the non-cycloplegic and cycloplegic measurements of the right eye obtained with 2WIN and ARK-1 devices. For non-cycloplegic measurements, the ARK-1 device scored a median ellipsoid of 1.00, and the 2WIN device scored 1.74 (Mann–Whitney z = 3.4, *p* < 0.01). For cycloplegic refraction with tropicamide, the ARK-1 device scored 1.43, and the 2WIN device scored 1.90 (Mann–Whitney *z* = 0.95, *p* = 0.340).

## Discussion

This study is the first to assess the correlation of non-cycloplegic and cycloplegic measurements between an autorefractor and a photoscreener. Its results showed a strong positive linear correlation for most of the examined parameters. Published studies have demonstrated that non-cycloplegic measurements obtained with photoscreeners are in between cycloplegic and non-cycloplegic refractometer measurements (Table 3). For example, Won et al. compared Plusoptix S09 measurements with non-cycloplegic and cycloplegic refractometry measurements and found a significant difference in SE among children aged 3–10 years (0.61 ± 2.02 D vs. −0.54 ± 1.98 D vs. 0.73 ± 2.05 D, respectively; *p* < 0.001) [10]. Similarly, Payerols et al. found a difference between PlusOptix A09 measurements and both non-cycloplegic and cycloplegic measurements (+0.54 ± 1.82 vs. 0.70 ± 3.14 vs. 1.06 ± 2.04) [11]. The difference between Plusoptix and cycloplegic measurements was significantly higher in hyperopes than in myopes (0.73 ± 1.34 vs. 0.05 ± 0.66; *p* = 0.010). Yakar et al. showed that non-cycloplegic measurements with the Spot were negative more often than cycloplegic measurements with the ARK-1 device (median: +0.25 vs. +1.12 D) [19]. The greater difference observed in that study could be partially explained by the fact that it was conducted on children aged 3–10 years.

**Table 3:** Selected studies comparing of spherical equivalent (SE) refraction between photoscreeners and other techniques in currently published studies

Study	Subjects	Design	Results (SE)
Liu et al. 2021 [20]	194 eyes of 97 children (age 4–14 years)	non-cycloplegic 2WIN vs. cycloplegic retinoscopy	−1.83 ± 1.48 vs. -1.38 ± 1.90 (*p* < 0.01)
Yakar et al. 2020 [19]	300 eyes of 150 patients (age 3–10 years)	non-cycloplegic Spot vs. cycloplegic ARK-1 refraction	+0.25 D vs. +1.12
Jesus et al. 2016 [12]	right eyes of 134 healthy participants (7–50 years)	cycloplegic Spot vs. subjective cycloplegic clinical refractometry	+0.66 ± 0.56 (*p* < 0.001)
Won et al. 2016 [10]	77 eyes of 40 children (2–10 years)	non-cycloplegic Plusoptix S09 vs. non-cycloplegic autorefractor vs. cycloplegic autorefractor (Canon RK-F1)	0.61 ± 2.02 vs. -0.54 ± 1.98 vs. 0.73 ± 2.05 (p < 0.001)
Payerols et al. 2016 [11]	70 eyes of 35 children (1–8 years)	non-cycloplegic Plusoptix A09 vs. non-cycloplegic vs. cycloplegic autorefraction (Nidek ARK-530A or Retinomax)	+0.54 ± 1.82 vs. -0.70 ± 3.14 (*p* = 0.04) vs. 1.06 ± 2.04 (*p* < 0.004)

Other studies examining the agreement in SE between photoscreeners and autorefractors/other measurement methods are presented in Table 3. This study found that the measurements obtained with the 2WIN device showed an excellent agreement both in non-cycloplegic and cycloplegic SE values. There was a trend towards more negative SE values obtained with the 2WIN device than with the ARK-1 device with increasing SE value; this was particularly evident for cycloplegic measurements. In hyperopes, the difference in SE was statistically significant for non-cycloplegic measurements (1.47 ± 1.42 vs. 2.29 ± 1.55 for the 2WIN and ARK-1, respectively) and even greater for cycloplegic measurements (1.22 ± 1.29 vs. 2.86 ± 1.71 for the 2WIN and ARK-1 devices, respectively).

Photorefraction has shown to be useful in screening large populations of children for refractive errors [21]. Despite the fact that photoscreeners have also been shown useful for adult examination [22], there are single studies evaluating the use of photoscreeners in adults.  It is a quick and relatively simple method that does not require the child’s active cooperation, making it suitable for young children who may have difficulty participating in standard eye exams. According to the American Association for Pediatric Ophthalmology and Strabismus the risk factors for amblyopia development include anisometropia greater than 1.5 D, hyperopia greater than 3.5 D, myopia greater than −3.0, with-the-rule or against the rule astigmatism greater than 1.5 D, and oblique astigmatism greater than 1.0 D [23]. The results of this study could confirm the utility of photoscreening for all these conditions; however, for hyperopes the results should be interpreted with caution.

The results of this study show an excellent positive correlation for the J0 (*r* = 0.92) and J45 (*r* = 0.83) magnitude, which was slightly lower for cycloplegic J0 (*r* = 0.67) and J45 (r = 0.78) values. The magnitude of astigmatism was not different between the devices for non-cycloplegic measurements (-0.75 ± 0.75 for 2WIN vs. -0.82 ± 0.81 for ARK-1; p=0.56); however, there was a difference for cycloplegic measurements (-0.56 ± 0.63 for 2WIN vs. -0.83 ± 0.78 for ARK-1; p<0.01). The pupil size variations may contribute to the differences observed in photorefraction values between measurements taken with and without cycloplegia; since vision screeners use a distant light source, aberrations associated with the larger pupil size may lead to slightly different refraction values. Other potential reasons include the differences in the measurement method and potential head tilt during measurements with the photoscreener. Published studies have reported similar results. Jesus et al. found a greater difference in the astigmatism value in the horizontal/vertical vector than in the oblique vector between the Spot device and subjective autorefractometry (+0.16 ± 0.27 [*p* < 0.001] and +0.02 ± 0.15 [*p* > 0.05], respectively) [12]. Won et al. showed that the Plusoptix S09 device significantly overestimates the cylinder value compared to non-cycloplegic and cycloplegic autorefractometry (−1.89 ± 1.63 vs. −1.34 ± 1.22 vs. -1.25 ± 1.20 D, respectively) [10].

In conclusion, cycloplegic refraction measurements obtained with the 2WIN photoscreener cannot be considered precisely interchangeable with those obtained with an ARK-1 stationary autorefractor. Nonetheless, a very strong correlation was found between the devices for most of the examined parameters.

## Data Availability

Data available on a reasonable request from the corresponding author (p.kanclerz@gumed.edu.pl )
